# Supplementary material for: The role of PilU in the surface behaviors of Pseudomonas aeruginosa
Source: mLife. 2025 Feb 23;4(1):83–95. doi: 10.1002/mlf2.12165 (PMC11868832; doi:10.1002/mlf2.12165)
Supplement: Supplementary file 1 — Supporting information. [file MLF2-4-83-s003.docx]

Supplementary Information for

**The role of PilU in the surface behaviors of *Pseudomonas aeruginosa***

Jingchao Zhang ^1^#, Yan Luo ^2,3^#, Yiwu Zong ^2^, Shangping Lu ^4^, Yi Shi ^5*^, Fan Jin ^6*^, Kun Zhao ^5,7*^

^1^Center for Medical Genetics, Sichuan Provincial People's Hospital, University of Electronic Science and Technology of China, Chengdu, Sichuan 611731, China.

^2^Frontiers Science Center for Synthetic Biology and Key Laboratory of Systems Bioengineering (Ministry of Education), School of Chemical Engineering and Technology, Tianjin University, Tianjin 300072, China.

^3^Guangzhou General Institute of Medical Research, Guangdong, Guangzhou 510000, China.

^4^School of Life Science and Technology, University of Electronic Science and Technology of China, Chengdu, Sichuan 610054, China.

^5^The Sichuan Provincial Key Laboratory for Human Disease Gene Study and The Institute of Laboratory Medicine, Sichuan Provincial People's Hospital, University of Electronic Science and Technology of China, Chengdu, Sichuan 610054, China

^6^CAS Key Laboratory of Quantitative Engineering Biology, Shenzhen Institute of Synthetic Biology, Shenzhen Institutes of Advanced Technology, Chinese Academy of Sciences, Shenzhen, Guangdong 518055, China.

^7^Institute of Fundamental and Frontier Sciences, University of Electronic Science and Technology of China, Chengdu, Sichuan 610054, China.

*Address correspondence to Kun Zhao, Fan Jin or Yi Shi

[kzhao@uestc.edu.cn](mailto:kzhao@uestc.edu.cn), [fan.jin@siat.ac.cn](mailto:fan.jin@siat.ac.cn;), or yshi@uestc.edu.cn.

^#^Jingchao Zhang and Yan Luo contributed equally to this work. Author order was determined by contributions in the order of presentation of data in the paper.

This PDF file includes:

Table S1

Figs. S1 to S6

Movie Legends S1 to S7

Table S1 Strains used in this study

| Strains | Characteristics | Source |
| --- | --- | --- |
| PAO1 wild type (WT) |  | Lab stock |
| ∆*pilU* | Cannot produce PilU | Lab stock |
| ∆*pilT* | Cannot produce PilT | Lab stock |
| ∆*pilA* | Cannot produce PilA | Lab stock |
| PAO1P*_BAD_*-pilAS99C (WT^m^) | Pherd-20T-pilA in PAO1 | This study |
| ∆*pilU*P*_BAD_*-pilAS99C (∆*pilU* ^m^) | Pherd-20T-pilA in ∆*pilU* | This study |
| ∆*pilT*P*_BAD_*-pilAS99C (∆*pilT* ^m^) | Pherd-20T-pilA in ∆*pilT* | This study |
| WT-GFP | GFP plasmid introduction into PAO1 | This study |
| Plasmids |  |  |
| pHERD-20T | pUCP20T with araC-PBAD, ^AmpR^ | This study |
| Pherd-20T-pilA | pUCP20T, ParaBAD-pilA(S99C), ^AmpR^ | This study |





Fig. S1. The fraction of motile cells in WT^m^ and ∆*pilU*^m^. The number of analyzed cell is *N* = 513 for WT^m^ and *N* = 343 for ∆*pilU*^m^. Statistical significances were measured using a two-sample Student’s *t*-test. * *p* < 0.05.





Fig. S2. Distributions of *γ*, which is defined as the angle between cell velocity and the x-axis. The number of analyzed cell is *N* = 5833 for WT^m^ and *N* = 5833 for ∆*pilU*^m^.


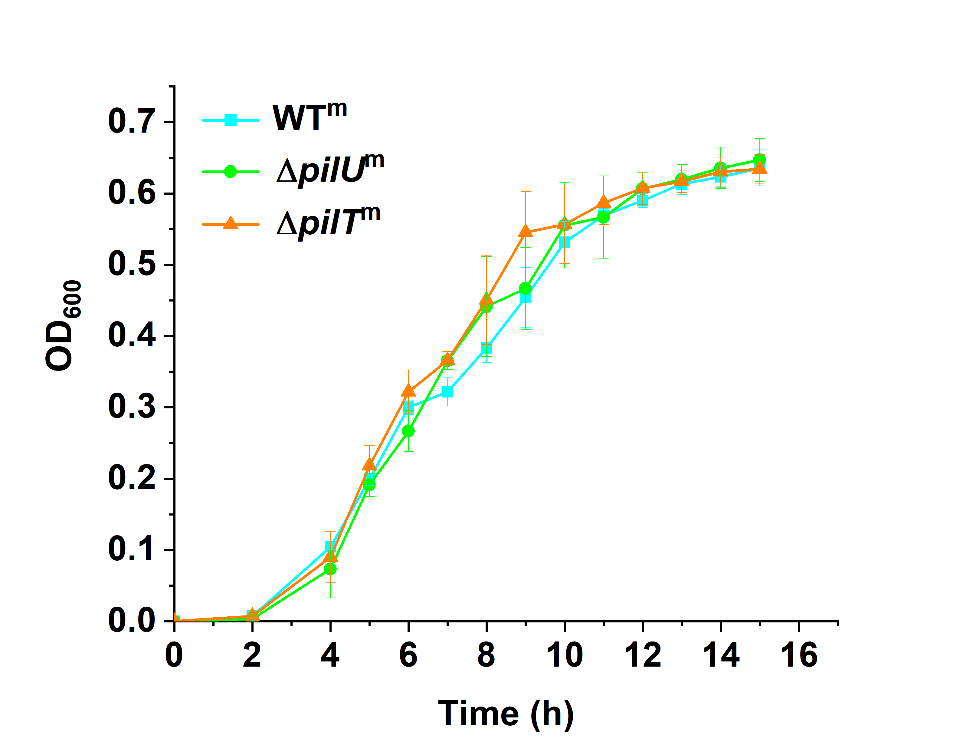


Fig. S3. Growth curves of strains used in this study in FAB medium containing 30 mM glutamate. WT^m^ (cyan squares); ∆*pilU*^m^ (green circles); ∆*pilT*^m^ (orange triangles). Error bars represent the standard deviation of three repeats.


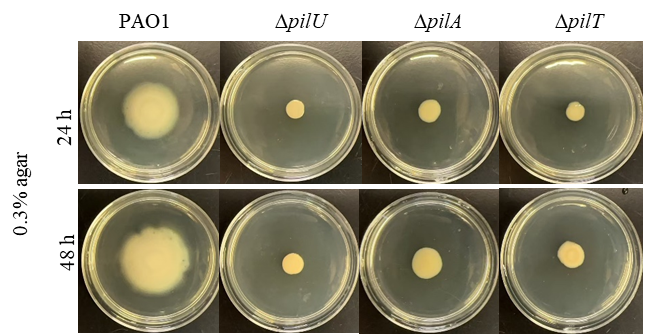


Fig. S4. WT and mutant expansion colonies on 0.3% agar surfaces.





Fig. S5. The fraction of stand-up bacteria at the edge of colony during the colony expansion of ∆*pilU*. The results were obtained from 11 fields of view, and the total count number of cells at the edge of colony is 496. Statistical significances were measured using a two-sample Student’s *t*-test. n.s., not significant.


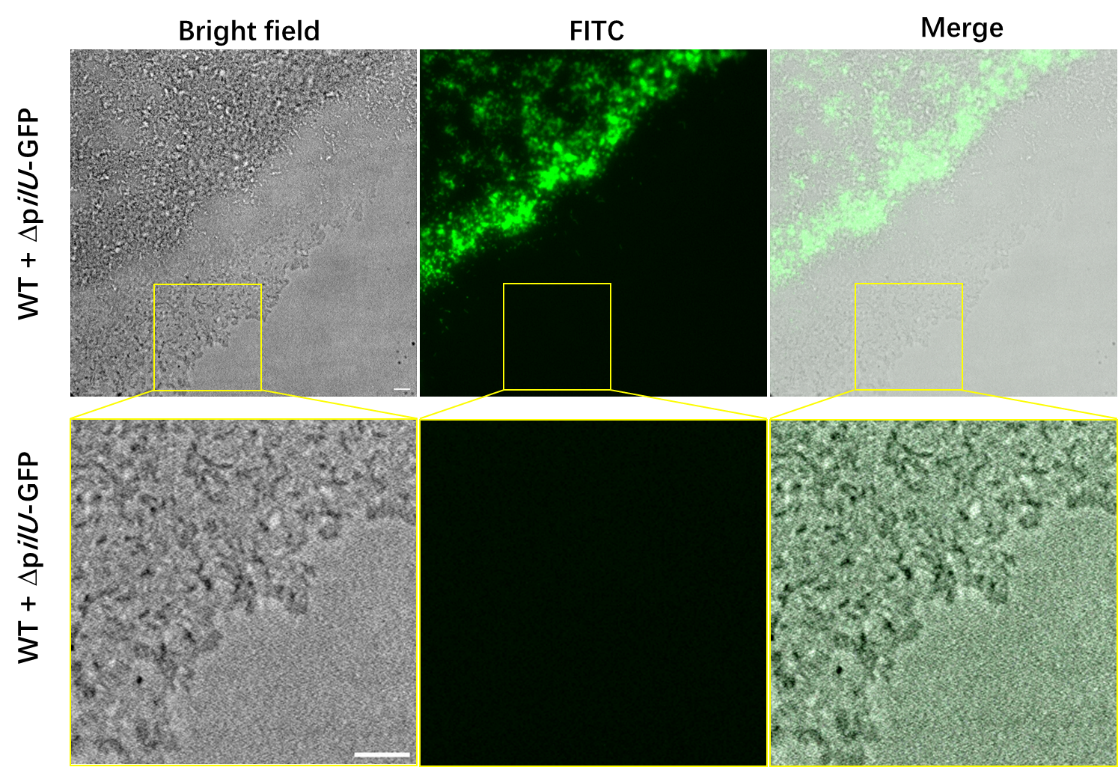


Fig. S6. Distribution of fluorescence signals in the expansion colony of WT + ∆*pilU*-GFP mixture. The images of the bottom row are magnified views of specific regions at the expansion edge marked by yellow squares.

Movie Legend

Movie S1. An example of bacterial surface movement of WT^m^. The movie was taken at a frame interval of 30 s and was played back at 5 fps. Scale bar of 5 μm shown.

Movie S2. An example of bacterial surface movement of ∆*pilU*^m^. The movie was taken at a frame interval of 30 s and was played back at 5 fps. Scale bar of 5 μm shown.

Movie S3. An example of bacterial surface movement of ∆*pilT*^m^. The movie was taken at a frame interval of 30 s and was played back at 5 fps. Scale bar of 5 μm shown.

Movie S4. A touch-turn example in WT^m^. The movie was taken at a frame interval of 2 min and was played back at 5 fps. The movie showed the merged results of bright-field and fluorescence images. Scale bar of 5 μm shown.

Movie S5. A touch-upright example in WT^m^. The movie was taken at a frame interval of 2 min and was played back at 5 fps. The movie showed the merged results of bright-field and fluorescence images. Scale bar of 5 μm shown.

Movie S6. A touch-turn example in ∆*pilU*^m^. The movie was taken at a frame interval of 2 min and was played back at 5 fps. The movie showed the merged results of bright-field and fluorescence images. Scale bar of 5 μm shown.

Movie S7. A touch-upright example in ∆*pilU*^m^. The movie was taken at a frame interval of 2 min and was played back at 5 fps. The movie showed the merged results of bright-field and fluorescence images. Scale bar of 5 μm shown.
